# Progress in COVID research and developments during pandemic

**DOI:** 10.1002/VIW.20210020

**Published:** 2022-07-20

**Authors:** Sudheesh K. Shukla, Santanu Patra, Trupti R. Das, Dharmesh Kumar, Anshuman Mishra, Ashutosh Tiwari

**Affiliations:** ^1^ Institute of Advanced Materials IAAM Gammalkilsvägen 18 Ulrika 59053 Sweden; ^2^ VBRI Innovation Centre 7/16 Kalkaji Extn New Delhi 110019 India; ^3^ CIPET, Institute of Petrochemicals Technology (IPT)‐Bhubaneswar Patia Bhubaneswar India

**Keywords:** artificial intelligence, corona virus, COVID science and technology, COVID‐19 diagnosis, pandemic years, respiratory tract infection, serological test

## Abstract

The pandemic respiratory disease COVID‐19 has spread over the globe within a small span of time. Generally, there are two important points are being highlighted and considered towards the successful diagnosis and treatment process. The first point includes the reduction of the rate of infections and the next one is the decrease of the death rate. The major threat to public health globally progresses due to the absence of effective medication and widely accepted immunization for the COVID‐19. Whereas, understanding of host susceptibility, clinical features, adaptation of COVID‐19 to new environments, asymptomatic infection is difficult and challenging. Therefore, a rapid and an exact determination of pathogenic viruses play an important role in deciding treatments and preventing pandemic to save the people's lives. It is urgent to fix a standardized diagnostic approach for detecting the COVID‐19. Here, this systematic review describes all the current approaches using for screening and diagnosing the COVID‐19 infectious patient. The renaissance in pathogen due to host adaptability and new region, facing creates several obstacles in diagnosis, drug, and vaccine development process. The study shows that adaptation of accurate and affordable diagnostic tools based on candidate biomarkers using sensor and digital medicine technology can deliver effective diagnosis services at the mass level. Better prospects of public health management rely on diagnosis with high specificity and cost‐effective manner along with multidisciplinary research, specific policy, and technology adaptation. The proposed healthcare model with defined road map represents effective prognosis system.

## Graphical abstract

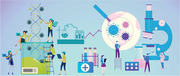

## 1 INTRODUCTION

Occurrence of the worldwide pandemic Coronavirus disease 2019 (COVID‐19) in later 2019 to early 2020 has twisted the human society through health crisis and creating new challenges to the world. Due to highly contagious nature, on March 11, 2020 the COVID‐19 virus outbreak has been declared as the global pandemic by World Health Organization (WHO). Since the first report of COVID‐19 at Wuhan, China in December 2019, it has been affected 225 countries and territories with 6,137,417 million confirmed death and close to 479,281,013 confirmed cases across the world, up to March 25, 2022.^1^, ^2^, ^3^ With this emergent crisis during pandemic year, science and technology tackles the way to report the hurdles for the COVID‐19 to mitigate the spread and progress of this disease. Science and technology is in concert part to diagnose and cure the COVID‐19 by different techniques.^1^, ^2^ The research on the COVID becomes very prevalent during pandemic years. From the search of the Scopus database with the keyword “COVID‐19” it resulted in 286,313 documents as collected on March 25, 2022.^4^

**FIGURE 1:**
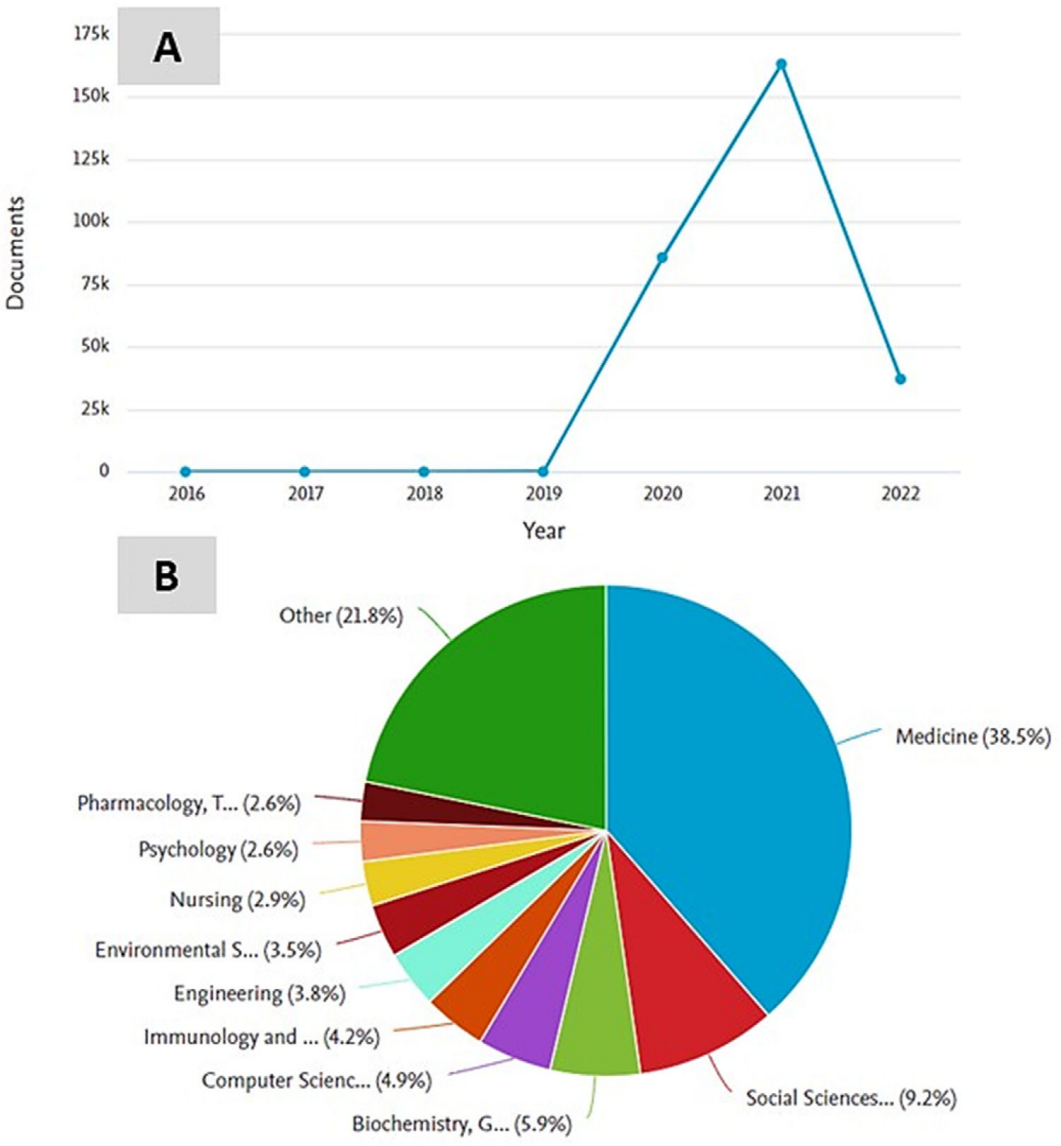
Documents on keyword “COVID‐19” indexed in the Scopus since 2006 to as on March 25, 2022. (A) Trend of documents published and (B) subject and field‐wise categories of the documents

The articles indexed in the Scopus show sudden trends of high publication in the literature on COVID, where the number of published articles get steep increased from 2019 (62 articles), 2020 (85,827 articles), and reaches 163,275 articles in 2021, then, there were 37,134 articles indexed even by first quarter of 2022 (Figure 1A). It is mainly due to the incident of COVID‐19 pandemic and research trends that were carried out in several domains of COVID including disease detection, diagnosis, infection monitoring, vaccine development, drug discovery, disease treatment, public health, government policies, etc. to control the biggest pandemic burden across the globe.

Moreover, the subject and field vise categories of the documents reveal that multidisciplinary research was reported mainly in Medicine (38.5%), Social Science (9.2%), Computer Science (5.9%), Biochemistry, Genetics and Molecular Biology (4.9%), and Immunology and Microbiology (4.2%) (Figure 1B). The type of indexed documents in Scopus emerges primarily research articles (64.6%), review articles (10.3%), letter (9.1%), conference (5.3%), etc. Further, COVID‐19 science and technology received vital directions at the global level, where many countries continuously contributed effectively in extensive COVID research and developments.

The corona virus is characterized by projecting spikes on its external surface, and it causes the pneumonia epidemic. The epidemic began from the city of Wuhan, China and then it prevailed all over the globe from in the end of 2019. During the pandemic year, it is identified that COVID‐19 is commonly related with acute respiratory infections in humans and cause respiratory and enteric infections, but it also has the capability to infect other associated species. Several factors such as disease prevalence, patient profile, sample type, disease stage, etc. are constraining the identification of COVID‐19 and are influencing the routine diagnostic test.^5^, ^6^ The common symptoms of COVID‐19 infection are mostly flu‐like. Severe lung infection also been identified at all ages, but high risk is associated with elderly patients. The COVID‐19 disease may lead to multi‐organ failure in human being by causing acute respiratory distress syndrome (ARDS). Again, it leads to severe interstitial pneumonia which ultimately resulting with severe acute respiratory failure and resulting with high death rates.^7^, ^8^, ^9^ In addition, COVID‐19 patients also display a radiological sign and variable extent of dyspnea. The reduction of rate of acerbating infections and decreasing of death rates have been focused and highlighted toward the diagnosis and treatment process.

In this systematic review article, R&D evidence of all the approaches during pandemic years using for screening and diagnosing the COVID‐19 infectious patient and additionally understanding the clinical and transmission models for early and advanced diagnosis, that is, the best perspective for clinical relevance were investigated. During this pandemic year, science and technology creates a standard diagnostic approach for the recognition of COVID‐19. The prospects of public health management depend on diagnosis with high specificity and cost‐effective manner along with multidisciplinary research, specific policy, research and development, and technology adaptation. Moreover, this paper focuses on the performance outcomes of key test and action deployed to diagnose and control COVID‐19 epidemics during pandemic years. Integration of several components of technologies such as sensor, artificial intelligence (AI) under framework of sustainable environment and socio‐economic aspects for diagnosis and preventive measures needed during pandemic years and our proposed sustainable healthcare model with defined road map represents the effective prognosis system.

## 2 BIOLOGICAL STRUCTURE AND TESTING INDICATIONS

**FIGURE 2:**
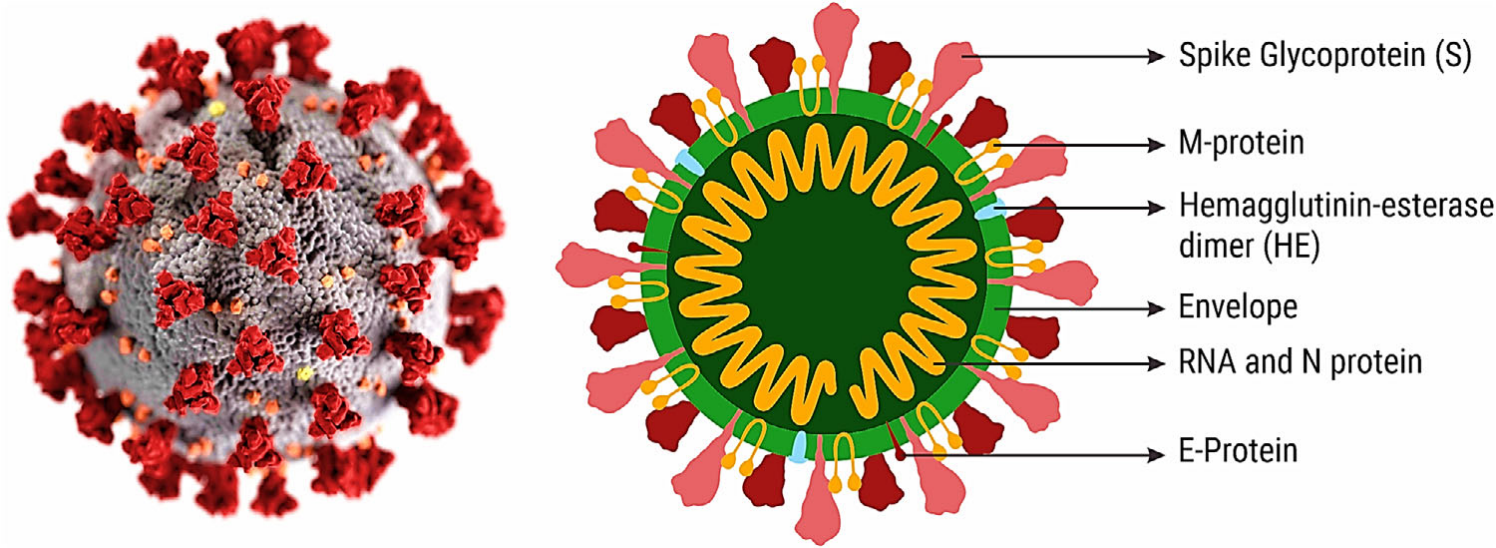
Composition of coronavirus. Microscopical structure of coronavirus defines the outer and inner components. Redrawn with permission^10^

Coronaviruses are with spherical surface and are called large pleomorphic spherical particles. 0.12 µm is the average diameter of the virus particle size and 0.08 µm is the diameter of the envelope. And the spikes have the length of 0.02 µm. The envelope of coronavirus looks like as a pair of distinct electron dense cells in electron micrographs. Membrane (M), envelope (E), and spike (S) structural proteins are the lipid bilayers found in the structural envelope of Covid 19, which are anchored to each other. A coronavirus with a shorter spike protein called beta‐coronavirus A and called hemagglutinin esterase. Nucleocapsid (N) proteins are found inside the envelope and bound to the RNA genome. The membrane proteins, nucleocapsid, and lipid bilayer envelop provide a shelter when virus is outside the host cell.^10^ The schematic presentation of coronavirus with entire structural segment has been displayed in Figure 2.

### 2.1 Clinical feature and pathological significance

The patient infected with corona virus shows long range of symptoms which includes asymptotic to septic shock, multi‐organ failure, and ARDS. In moderate cases, COVID‐19 infected patient shows a common symptom such as cough, sore throat, fever, shorten of breath, malaise, and headache, while in severe cases patients may suffer from ARS, septic shock, and pneumonia like symptoms. Laboratory finding for the COVID‐19 patients with abnormal state of myocardial, C‐reactive protein, creatine kinase, lactate dehydrogenase, lymphopenia, along with elevated interleukins (ILs), tumor necrosis factor‐alpha (TNF‐ α), prothrombin time, and renal or hepatic injury. The spectrum of the available diagnostic tools is limited for corona virus; therefore, science and technology has been a huge challenge to resolve this pandemic situation. During pandemic year, several commercially available multiplex tests for the identification of COVID‐19 via respiratory routes are employed. As the epidemics progresses in pandemic years, country‐specific signs and standards from testing have been changed rapidly. By clinical point‐of‐view, diagnosis for the COVID‐19 should be focused with person has an infection in acute respiratory tract with or without systemic symptoms. While, in case of mild symptoms, diagnosis directing the necessity quarantine and pinpointing of new cases throughout the method of contact tracing and testing of the contact.^11^, ^12^

**FIGURE 3:**
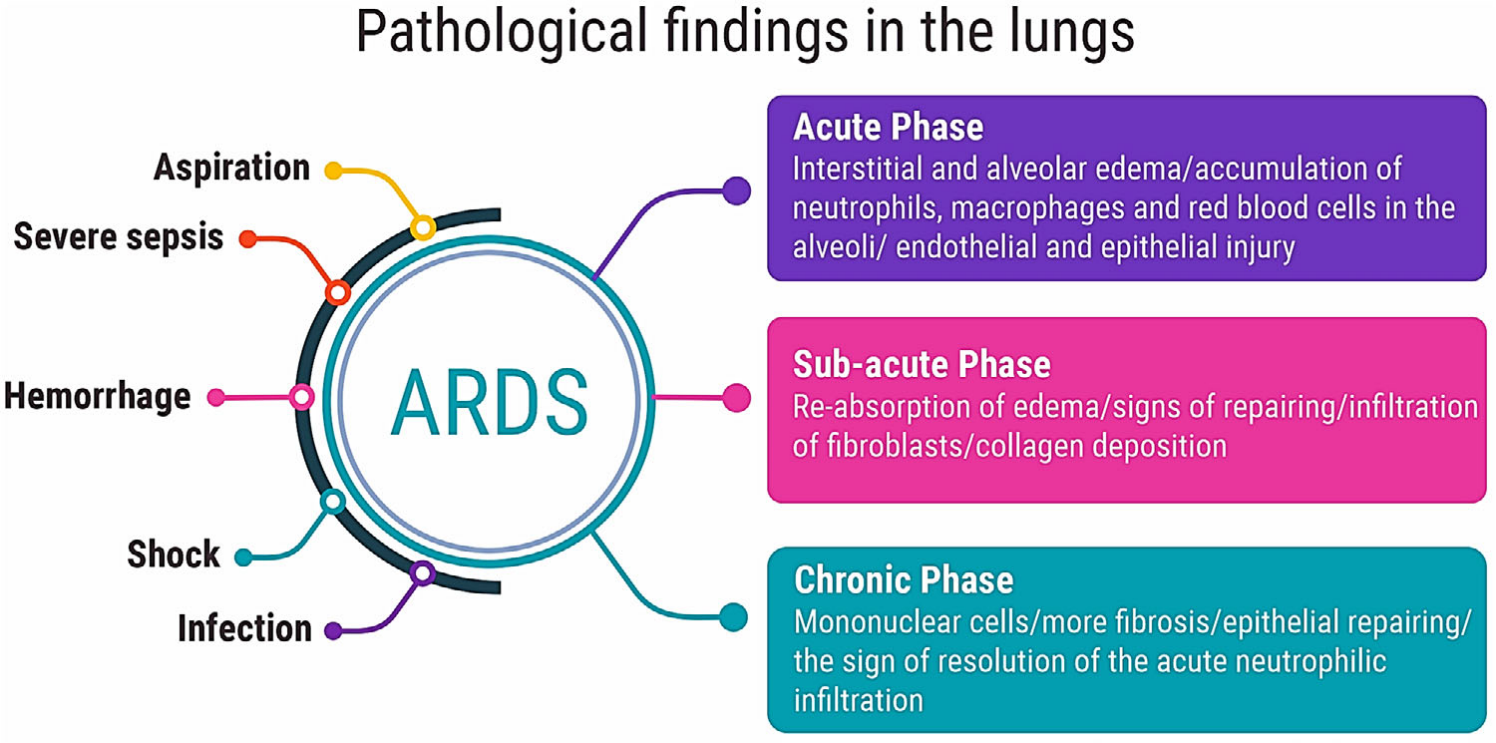
Pathological findings in the lungs. Acute respiratory distress syndrome (ARDS)

As per the severity of the infection, the typical pathological symptoms appear in three main phases, that is, *acute*, *sub‐acute*, and *chronic phase*. The first 6 days of the infection refers to the acute phase, where alveolar and interstitial edema with the accumulation of red blood cells (RBC), macrophages, and neutrophils have been seen, whereas the sub‐acute phase of infection characterized by the infiltration of fibroblast and collagen deposition. The sign of repairing and re‐absorption of edema could be seen in the second week. While, after second week the sign of tenacity of the acute neutrophilic infiltration, more number of mononuclear cells and more fibrosis and epithelial repairing could be found, and this phase is known as the chronic phase of COVID‐19 infection.^13^ The ray‐diagram of all these three phases of infection has been presented in Figure 3.

Due to the ARDS, severe sepsis, aspiration of gastric contents, pulmonary or non‐pulmonary infection hemorrhage, and shock disease arise. There are three groups of pathological findings of ARDS, and it depends on the phase of occurrence: acute phase, sub‐acute phase, and chronic phase.^13^

## 3 BIOLOGICAL SAMPLING AND ASSAY FOR COVID‐19 DIAGNOSIS

**FIGURE 4:**
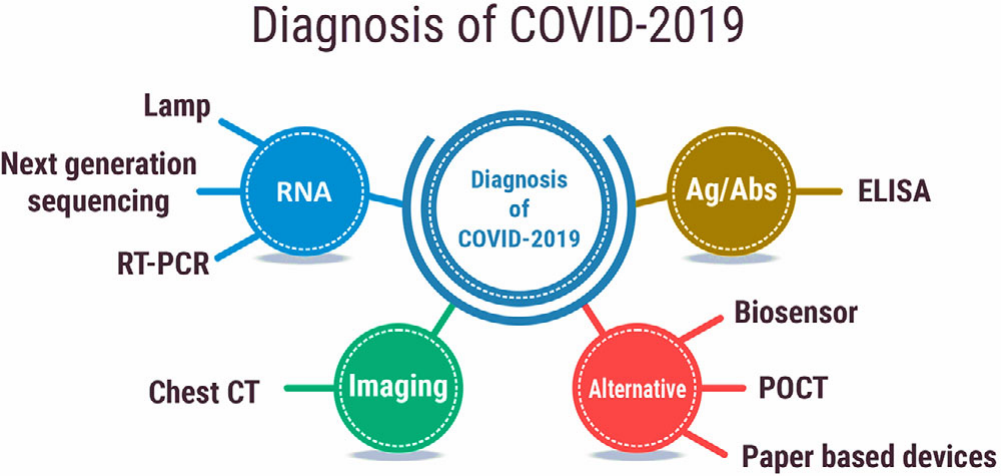
Diagnostic approaches for COVID‐19. Redrawn with permission^14^

The accurate and quick diagnosis of COVID‐19 infection is significant to control disease by initiating public healthcare systems. During pandemic year, there are several biological assays ranging from molecular to immunological detection used for early diagnosis, but important diagnostic approach based on the travel history as well as clinical symptoms along with some auxiliary medical examinations. In case of COVID‐19, symptoms like pneumonia are very atypical and relatively similar. At the initial stage of pandemic year, the rapid diagnostic method for COVID‐19 was unclear but at the later stage, research and development employed very useful diagnostic methods are currently in practice (Figure 4).

Understanding of the different protein targets and its respective potential drug candidates along with bioassay and structure–activity association data for the coronavirus virus infections playing an important role effective solution for COVID‐19 disease health management.^15^, ^16^Either the amount of SARS‐CoV‐2 in patient depends on the time of infection and different sources of sample such as sputum, stools, urine, and nasopharyngeal swab, but nasopharyngeal and oropharyngeal sample obtained with a swab is the most common sample for the testing of COVID‐19 by means of reverse transcription polymerase chain reaction (RT‐PCR). Dry swab is more conducting and less costly for the community‐based testing. Bronchial washing, tracheal aspirate, broncho alveolar lavage, and sputum are the samples from the lower respiratory tract, also been used for the testing of COVID‐19, whereas samples from the lower respiratory tract have more possibility to test positive. In case of COVID‐19's, viral ribonucleic acid (RNA) can also be diagnosed in blood (∼1%), in stool (∼30) but rarely in the urine sample.^17^ Depending on the recent data from the infections, patients with mild symptoms (80%–90%) not requiring hospitalization, while the 10%–20% symptomatic patients require hospitalization due to previous health complexity (immune‐compromising illnesses, tobacco smoking history, respiratory complications), and age (> than 50 years) factor. In case of mild disease at early stage of infection, RT‐PCR can show ∼30%–40% false negative report and in this case, patient needs to undergo five repeated tests to confirm a positive report. Owing to the limit of sensing of the assay, low‐sample volume, sampling location, sample‐processing methodology, timing of the sampling with respect to the stage of the disease and low viral load diagnosis of false negative results have been ascertain.^18^, ^19^, ^20^, ^21^ The entire list of viral diagnosis tools has been concise in Table 1.

**TABLE 1:** Current diagnostic methods available in practice for the COVID‐19

S. No.	Diagnostic tests	Assessment methods	Ref.
1.	Immunoassays	Fluorescent antibodies, Staining Hemagglutination, Immuno‐peroxidase staining, EIA/ELISA	22, 23
2.	Mass spectrometric methods	MALDI‐TOF	24, 25
3.	Direct visualization of virus	Electron microscopy	26, 27
4.	DNA sequencing	DNA microarray, Sanger sequencer, Next‐generation sequencer	28, 29, 30, 31
5.	Nucleic acid detection and amplification	PCR, RT‐PCR, qPCR, Isothermal amplification technologies (RCA, LAMP, NEAR, TMA, SDA, and HDA)	32, 33, 34, 35
6.	Microelectronic‐ and microfluidics‐based techniques	Point‐of‐care testing, Surface Plasmon resonance technique, Lab‐on‐chip testing	36, 37, 38

Abbreviations: ELISA, enzyme‐linked immunosorbent assay; MALDI‐TOF, time of flight; PCR, polymerase chain reaction; RT‐PCR, reverse transcription polymerase chain reaction; qPCR; RCA, rolling circle amplification; LAMP, loop‐mediated isothermal amplification; NEAR, TMA, transcription‐mediated amplification; SDA, strand displacement amplification; HDA, Helicase‐dependent amplification HDA.

## 4 COVID‐19 BIOMARKERS AND BIORECOGNITION ELEMENTS

As an indicator for the COVID‐19 diagnosis numerous biomarkers and antigen are identified. At the beginning of pandemic years, the viral genetic materials extracted from the nasopharyngeal swab and sputum which are later tested by the RT‐PCR, which have the maximum level within 5–6 days after symptoms inception with viral load of ∼10^4^–10^7^ copies ml.^1^ ^39^ ^‐1^ ^42^ But currently, COVID diagnosis based on the detection viral biological markers, that is, viral protein (S and N proteins) and viral genetic materials (RNA).^43^ In addition, the host immune responses to COVID‐19 infection are one of the other indicators for diagnosis. Thus, human immunoglobulins (Igs) with biorecognition elements (antigen (Ag)‐like molecules, antibodies (Abs), and nucleic acid probes) that is explicitly attach to biomarkers for the SARS‐CoV‐2 diagnosis.^44^, ^45^

Currently, COVID‐19 virus RNA (standard recognition molecule is nucleic acid probes) used as a major biomolecule for the proof of identity. The whole genome of SARS‐CoV‐2 could be identified by designing the SARS‐CoV‐2 specific primers to amplify a unique sequence of SARS‐CoV‐2 genome by using RT‐PCR.^46^, ^47^ Second, the N and S protein behave as the antigen as well as biomarkers for SARS‐CoV‐2 recognition, while the Abs or Abs‐like molecules act as their biorecognition molecules. The S‐protein bind with host ACE2 to enable the passage of SARS‐CoV‐2 into the respective cell. While N‐protein is a multifunctional RNA‐binding protein engage with replication and assembly along with the expression at the high level in infected cell at the early stage of COVID infection.^48^, ^49^, ^50^

## 5 BIOCHEMICAL AND BIOPHYSICAL ASSAY

### 5.1 RT‐PCR assay

In current scenario, the RT‐PCR method is most reliable diagnosis technique for COVID‐19. The genetic information (i.e., RNA) about the corona virus is noticed by the RT‐PCR. It is possible only when if the virus is available and someone actively infected. The presence of antigen (Ag) of the COVID‐19 is determined by the PCR test, directly. Sequencing of coronavirus has facilitated molecular detection assay and the entire assay involve in two fundamental steps: (1) extraction of viral RNA from patient swab and (2) RT‐PCR amplification by using specific primers and specific probe. The implementation of the robotic system facilitates the RT‐PCR to increased throughput for PCR set up and RNA extraction, the entire RT‐PCR processes have shown in Figure 6. For entire process at least two‐target gene is essential for confirming the presence of COVID‐19 in the patient sample. On the laboratory point of view, multiplexing of the target allows the better efficiency with short time span.^51^

Advancement with RT‐PCR, digital PCR (dPCR) has been implemented to achieve the excellence confirmation. By using the dPCR approach, 28.2% sensitivity significantly can be improved. The diagnostic accuracy, overall specificity, and sensitivity of RT‐dPCR were reported as 100%, 93%, and 90, respectively. In comparison to conventional RT‐PCR, RT‐dPCR has higher sensitivity for detection of viral RNA. In addition, RT‐dPCR is much preferable for recognition of cost implication, lower viral load, inability to multiplex target genes of interest, and limited complexity.^52^ For emergency purpose, the Food and Drug Administration (FDA) has received automated rapid nucleic acid amplification test proposals. Among them, the Cepheid's Xpert Xpress runs on the Gene Xpert platform can detect multiple gene target and gives the entire information within 45 min. Again, the Abbott's rapid COVID‐19 test which runs on Abbott ID NOW device provides the results within 13 min.^53^, ^54^

### 5.2 Immunoassay

**FIGURE 5:**
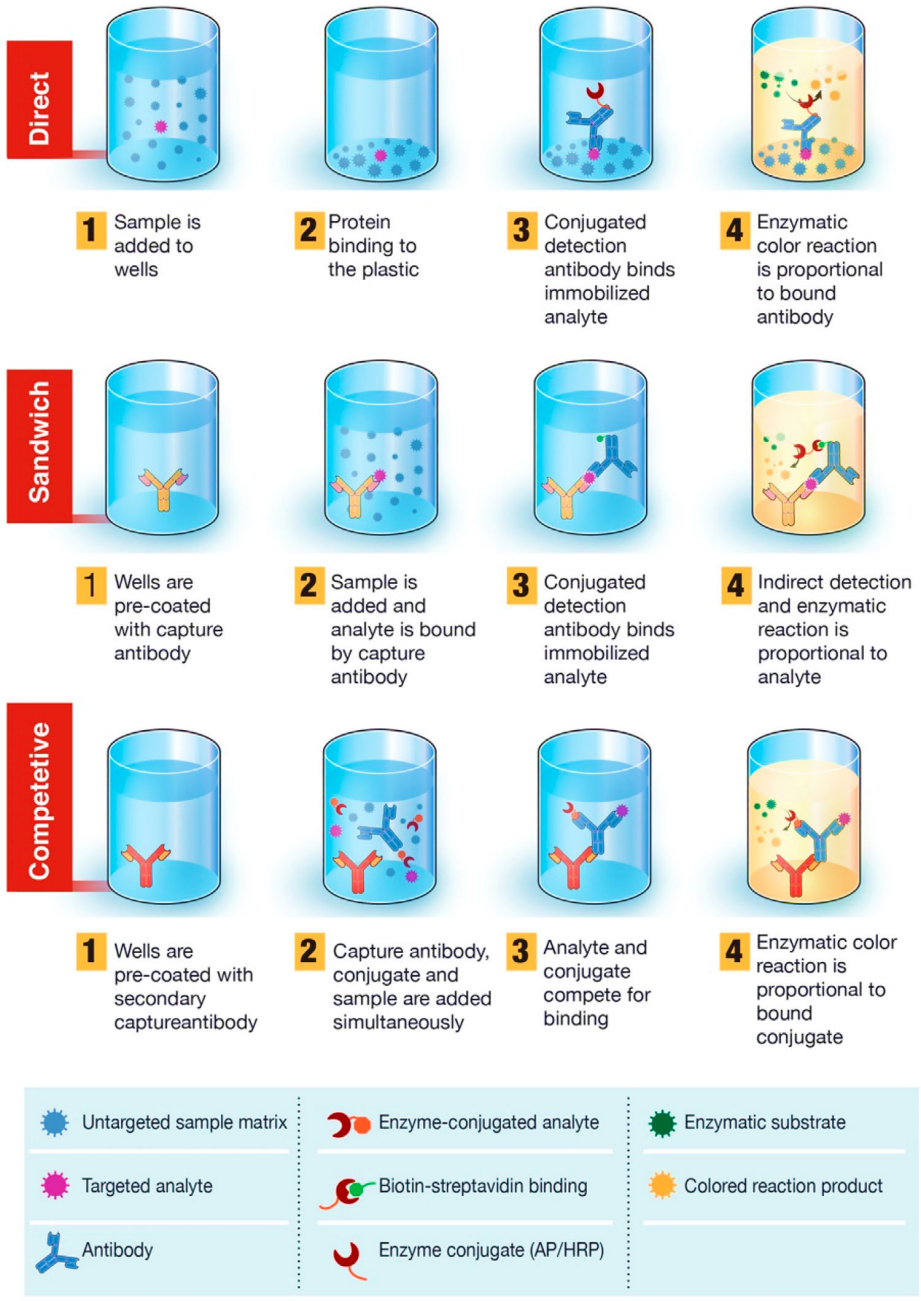
Basic principle of enzyme‐linked immunosorbent assay (ELISA) serological tests. Here blood sample, that is, plasma is applied to antigen‐coated microplates. On the coated antigen surface, anti‐SARS‐CoV‐2 antibodies are immobilized. Then, it follows the detection conjugate and color devolvement. A chronic reaction gets catalyzed with the binding of enzyme‐coupled secondary antibody to the immobilized antibody. Reproduced with permission^56^

Ag‐based immunoassays tests have been developed for recognizing of Abs in the plasma or serum. Recently, FDA approved one of the Ag‐based technology namely Foundation for the Innovative New Diagnostics from the list of 200 companies. All these technologies are dependent on either lateral flow assay (LFA) or enzyme‐linked immunosorbent assay (ELISA) like test. The mechanism involved in both of the tests is with antigen impregnated on a plastic‐plate surface or a test line, where human immunoglobin, IgG, IgM, and sometime IgA find and ultimately detection cause. Whereas for the time being, a rapid capture assay in nasopharyngeal aspirates have been developed which detect viral Ag are being evaluated. Notwithstanding, propagation of different testing kits and devices are barely any independent data validation on which optimally specific assay execute. Hence, the specificity, sensitivity, and predictive value of individual test are still anonymous; while some of them are web‐based resources that have been approved only in limited countries^55^. Serological ELISA test based on lateral flow test and an automated which allow high throughput screening for the hundred patients at a time. The ray‐diagram of serological ELISA test is shown in Figure 5.

Several researches described that longitudinal Ab response in COVID‐19 patients, where IgM responses lean to become traceable within 3–7 days of the infection, while the robust response of the infection generally seen in by the second week of the infection. Due to these deliberation, the Ab‐based test are suggested for front line of diagnosis at the beginning stage of infection but combining the Ab‐based assay and RT‐PCR data during early phase of diagnosis giving the fruitful conclusion. In this content, WHO's advisory recommending that serological testing is not suitable for the diagnosis of acute COVID‐19 infection, but it can be limited for the epidemiological investigation.^57^, ^58^, ^59^ Out of these, there is an undeniable role for immunoassays in diagnosis of COVID‐19 patient especially with respect to guide the public's health planning and in defining the route of the epidemics. Some of the research suggested that immunoassays could be helpful to identify the early infected and re‐covered health care workers along with other essential first line warrior of COVID‐19. Weather it is un‐clear that for how long the immunity can last against COVID‐19, therefore the valedictory concept of immunity passport is remains under investigation.^60^

## 6 TECHNOLOGY‐BASED ASSESSMENTS

In current pandemic health crisis, the healthcare sector looks for the technology assessment approach to monitor and control the early screening, control, and spread of COVID‐19. Technologies have the potential to enhance the public health control management extensively. Inclusion of various technology‐based solutions is more useful for the public healthcare management. Technology‐based assessment is an essential to get the effective results for COVID‐19 control. During the COVID‐19 pandemic, the technology‐based environment has taken a paradigm shift in diagnosis method of assessment for fast, reliable, and real‐time manner. This work describes the several of technology‐based assessment and their application in COVID‐19 public health management.^61^

Presently, efforts have been continuously made to understand the appropriate diagnostics, therapy, and monitoring. Recently, international health agencies such as Center for Disease Control (CDC) and WHO have requested to experts to form the bio‐medical companies, research institute, and universities to accelerate the efforts for the primary level detection of COVID‐19. According to CDC and WHO, RT‐PCR is the available method for COVID‐19 diagnosis. The RT‐PCR is an effective diagnostic procedure to detect COVID‐19.^62^

### 6.1 Biosensor‐based promising approach

Out of all the technology‐based assessment, biosensor is one of the front‐line techniques for the diagnosis of COVID‐19. To overcome pandemic challenge, there is a need to design and develop a smart sensor. Due to advancement in genetic engineering, nanotechnology, and transduction system, various strategies improve the detection performance of biosensor.^63^, ^64^, ^65^

**FIGURE 6:**
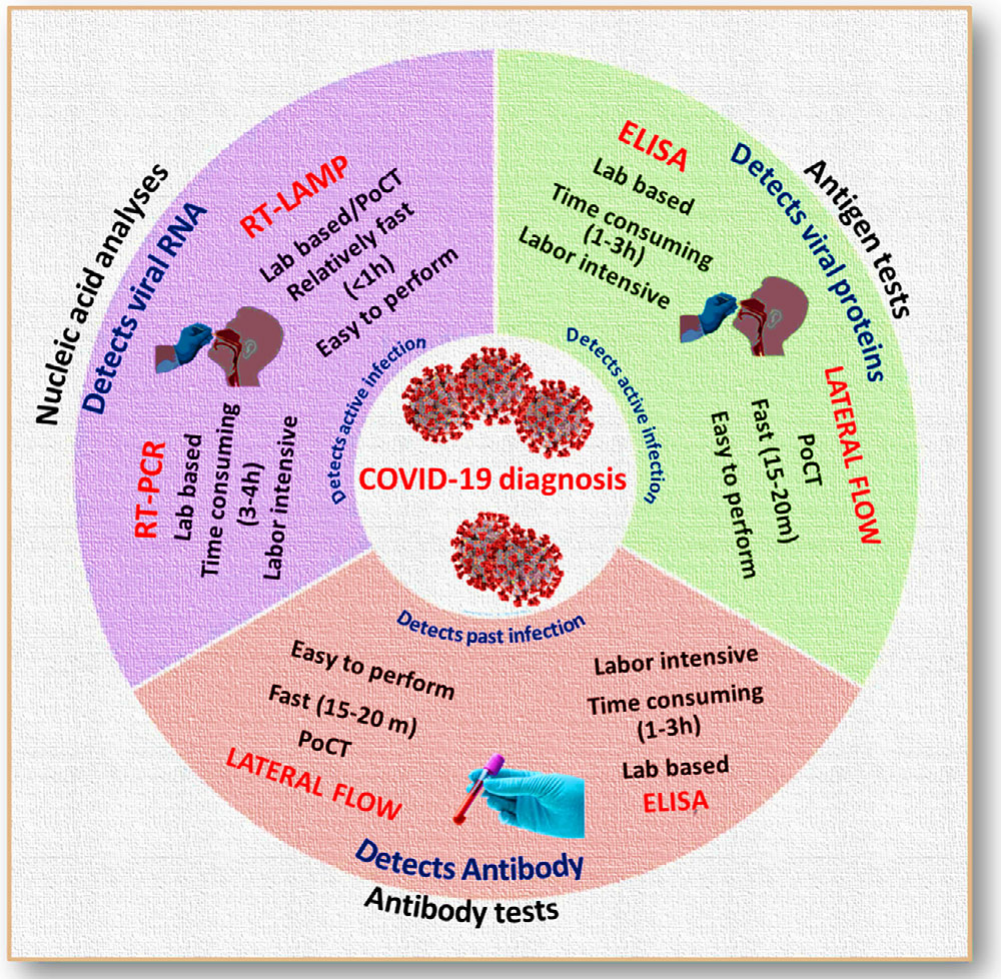
Various methods for COVID‐19 diagnosis. Abbreviations: PoCT, point of care test; RT‐LAMP, reverse transcription loop‐mediated isothermal amplification; RT‐PCR: reverse transcription polymerase chain reaction). Reproduced with permission^67^

The introduction of nanotechnology aids sensing of SARS and MERS at an exceptionally trace level.^66^ All the possible targets of the COVID‐19 diagnosis are membrane protein, glycoprotein, and viral genomic RNA which insist on instant immune response upon binding with host angiotensin‐converting enzyme‐2 (ACE‐2) receptors, while the humoral response is refered by immunoglobulin M (IgM) and immunoglobulin G (IgG) Abs. These factors are used to detect by the biosensor approach for diagnose the COVID‐19 and used in case of possible therapy known as plasma therapy (PT).^67^ The schematic presentation of COVID‐19 and its possible targets for diagnosis are represented in Figure 6. To overcome the conventional methods (i.e., ELISA, LFA, and colorimetric assay) issues (low accuracy and time consuming), researchers developed a rapid, affordable, and highly sensitive device to detect the COVID‐19. To shoot out the limitation of the qRT‐PCR based assay, extremely specific reverse transcription loop‐mediated isothermal amplification (RT‐LAMP) based assays have been developed for the recognition of COVID‐19. Recently, Zhu et al. developed single‐step RT‐LAMP mediated nanoparticles‐based biosensor for accurate and rapid diagnosis of COVID‐19.^68^

### 6.2 Immuno‐sensor technology

Recently, different types of biosensors such as CRISPR (Clustered Regularly Interspaced Short Palindromic Repeats), FET‐Graphene, Au‐nanoparticle/FTO, SPR‐based biosensors have been advanced for the detection of COVID‐19.^69^, ^70^, ^71^ Recently, various host biomarkers such as CRP, erythrocyte sedimentation rate (ESR), neutrophil lymphocyte ratio (NLR), procalcitonin (PCT), IL‐6 and biochemical (i.e., D‐dimer, troponin, creatine kinase, aspartate aminotransferase), lymphocyte count, neutrophil count, and especially related to coagulation cascade are identified from human host.^72^

**FIGURE 7:**
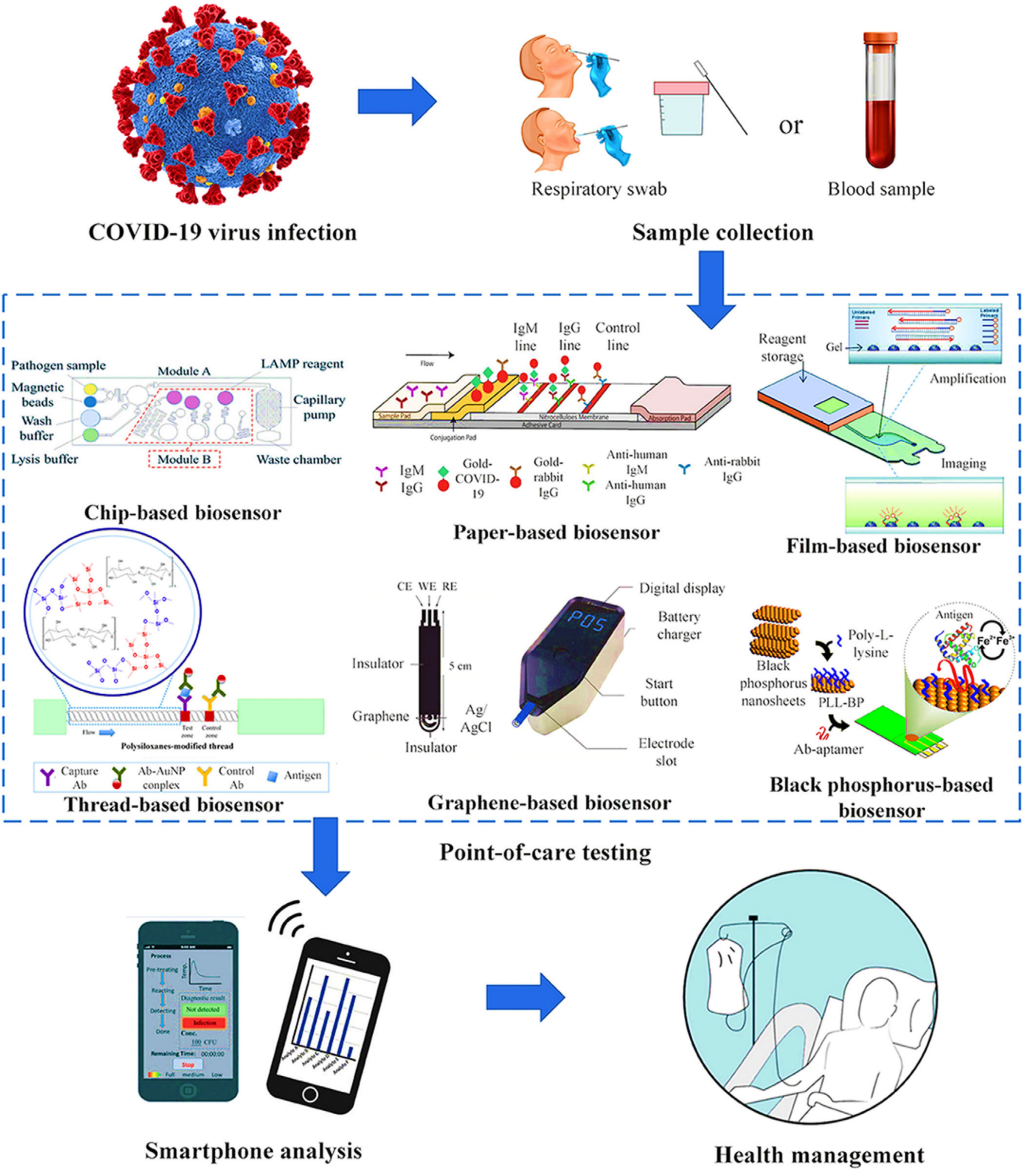
Biosensors for SARS‐CoV‐2 virus detection. Reproduced with permission^73^

Recently, Qiu et al. reported 2D gold nano‐island (AUNIS) functionalized cDNA receptors, based on a hybrid sensing mechanism of PPT effect and LSPR to provide an alternative and favorable solution for the detection of COVID‐19. This developed COVID biosensor exhibits a precise detection of the specific target for SARS‐CoV‐2 sequences in a multi‐gene mixture.^73^, ^74^ Figure 7 shows the different developed biosensor approach till date for the detection of COVID‐19.^73^ Conversely, either biosensor approach is under trial version and not in stage to fulfill the new viral challenges such as rapid mutation but in near future the biosensor‐based technology could pave the efficient way for rapid, sensitive diagnostic devices for the COVID‐19, and other unpredictable pandemics.

**FIGURE 8:**
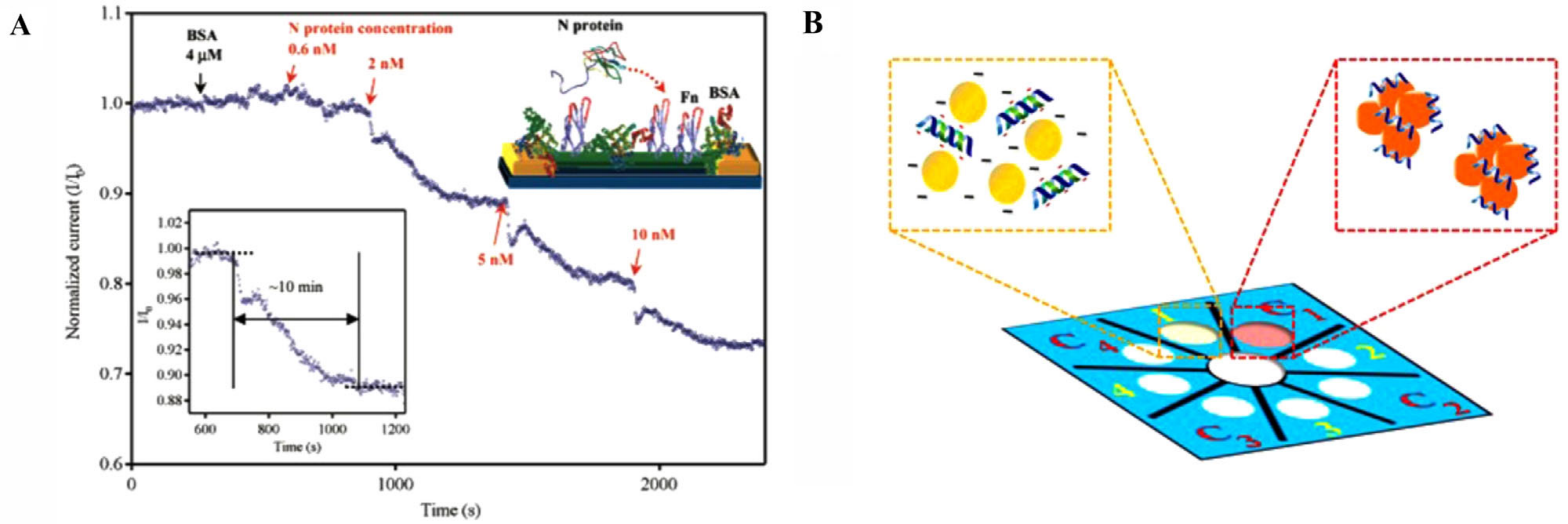
(A, B) Schematic presentation for the sensing performance for the COVID‐19 diagnosis via selective detection. Reprint with permission^76^, ^77^

Recently, this developed piezoelectric sensor detected SARS for long term in the short period of time.^75^ In current scenario, nanoscience and nanotechnology playing an important role improve the sensing performance and represent best option for COVID‐19 diagnosis via virus protein selection. For same, smart nanostructures have been encouraged to fabricate a biosensor which are electroactive in nature. A nanowire‐ based label‐free electrochemical sensing of the SARS Virus N‐Protein using an antibody mimic protein (AMP) approach is fabricated by Ishikawa et al. (Figure 8A).^76^ Teengam et al. established a paper‐based colorimetric assay for diagnosis of various pathogens DNA (Figure 8B).^77^ This study also revealed that the developed system is also effective for multiplexed detection (in optimized case) for other viruses too such as Zika and Ebola.^77^

**FIGURE 9:**
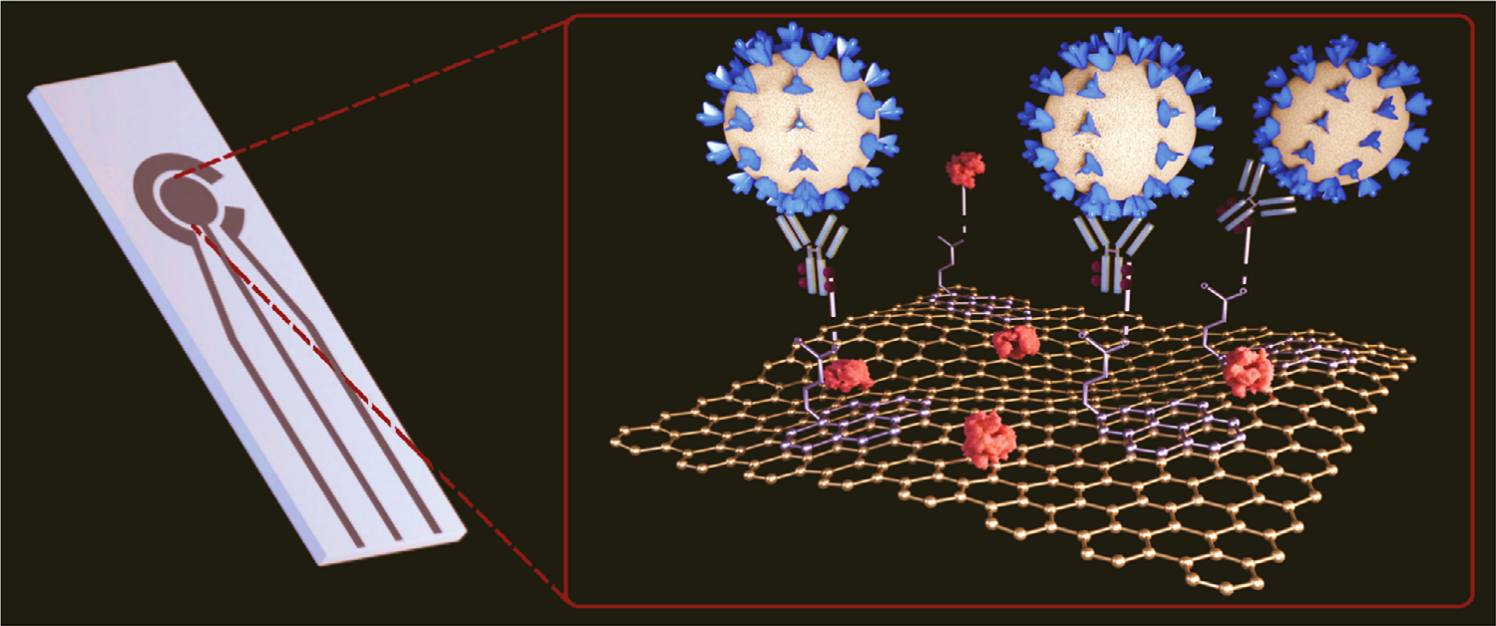
Electrochemical immunosensor for rapid detection of coronavirus. Reproduced with permission^79^

**FIGURE 10:**
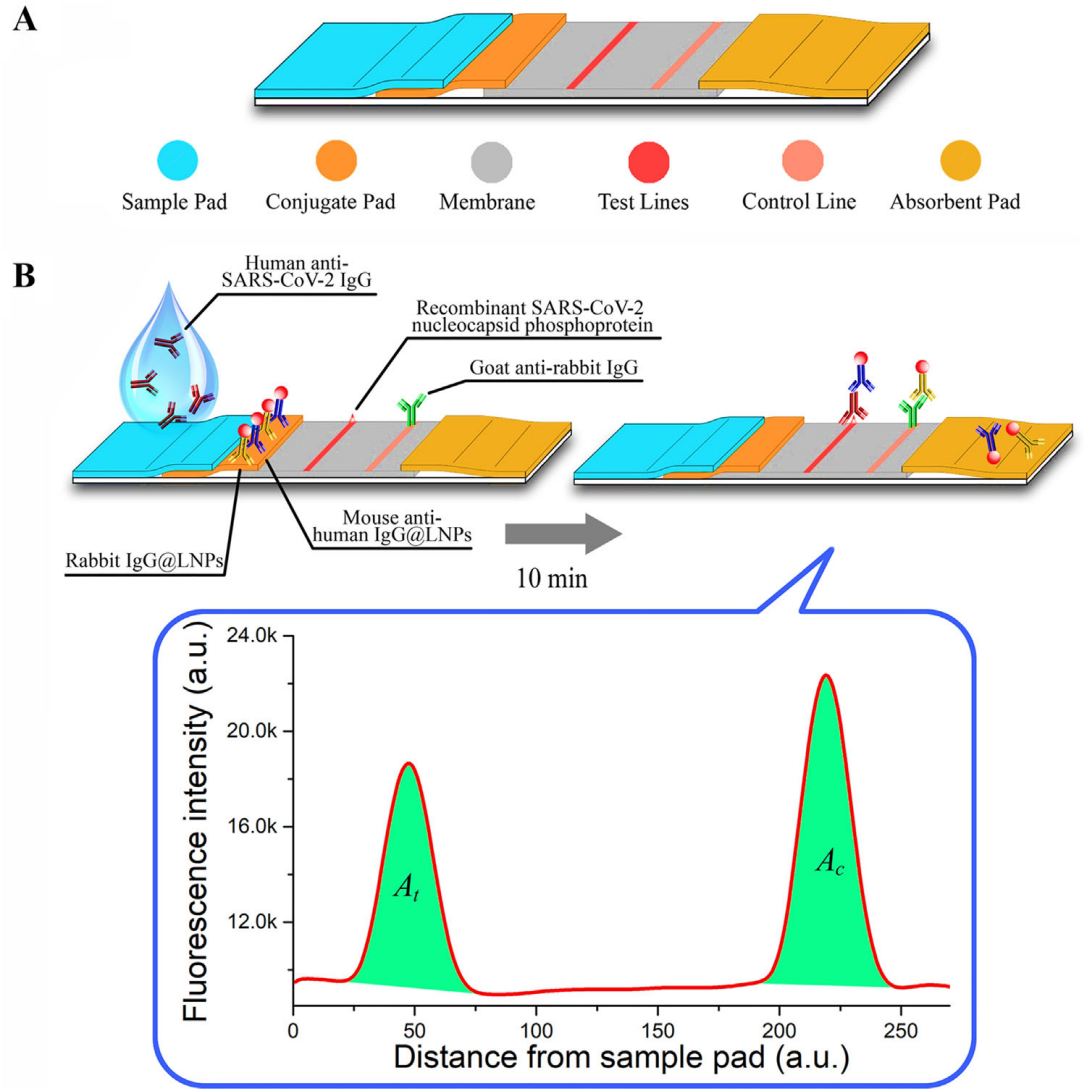
Rapid and sensitive recognition of anti‐SARS‐CoV2 IgG using Lanthanide‐doped nanoparticles‐based lateral flow immunoassay. Design and fabrication of the developed assay; and (A) lateral flow test strip and (B) assay test. Reprint with permission^80^

The MoS_2_ nanosheet was applied in practice to develop a fluorescent immunosensor for the detection of an avian COVID, that is, infectious bronchitis virus.^78^ Real sample of chicken serum was considered for the verification of the developed sensor. On the cheaper cotton‐thread‐based microfluidic manifold, this flexible optical sensor was fabricated. However, further validation for human's diagnostics needed. In case of COVID‐19, it is also reported that it has mutated and exhibited various strains depending on region, race, and country. Thus, diagnosis of selective COVID‐19 strains is the challenging aspect. Keeping in mind these challenges, Broughton et al. have detected SARS‐CoV‐2 virus protein selectively within 40 min by designing a LFA using the CRISPR−Cas12 gene. The CRISPR‐based test is also one of the best options for pathogen detection with high sensitivity and selectivity. CRISPR‐based design of the Cas‐12 gene is very suitable for point‐of‐care (POC) applications.^69^ The combinational sensing approach for diagnostics of COVID‐ 19 is also useful. Figure 9 shows the use of graphene for the detection of COVID‐spike protein.^79^ The portability of the fabricated sensor suggested its onsite application. However, more supportive studies and validations required. Chen et al. developed a lateral flow immunoassay (LFIA) fabricated using a lanthanide‐doped polystyrene Nano system for human diagnosis^80^ (Figure 10). However, more samples are required for validity.

**FIGURE 11:**
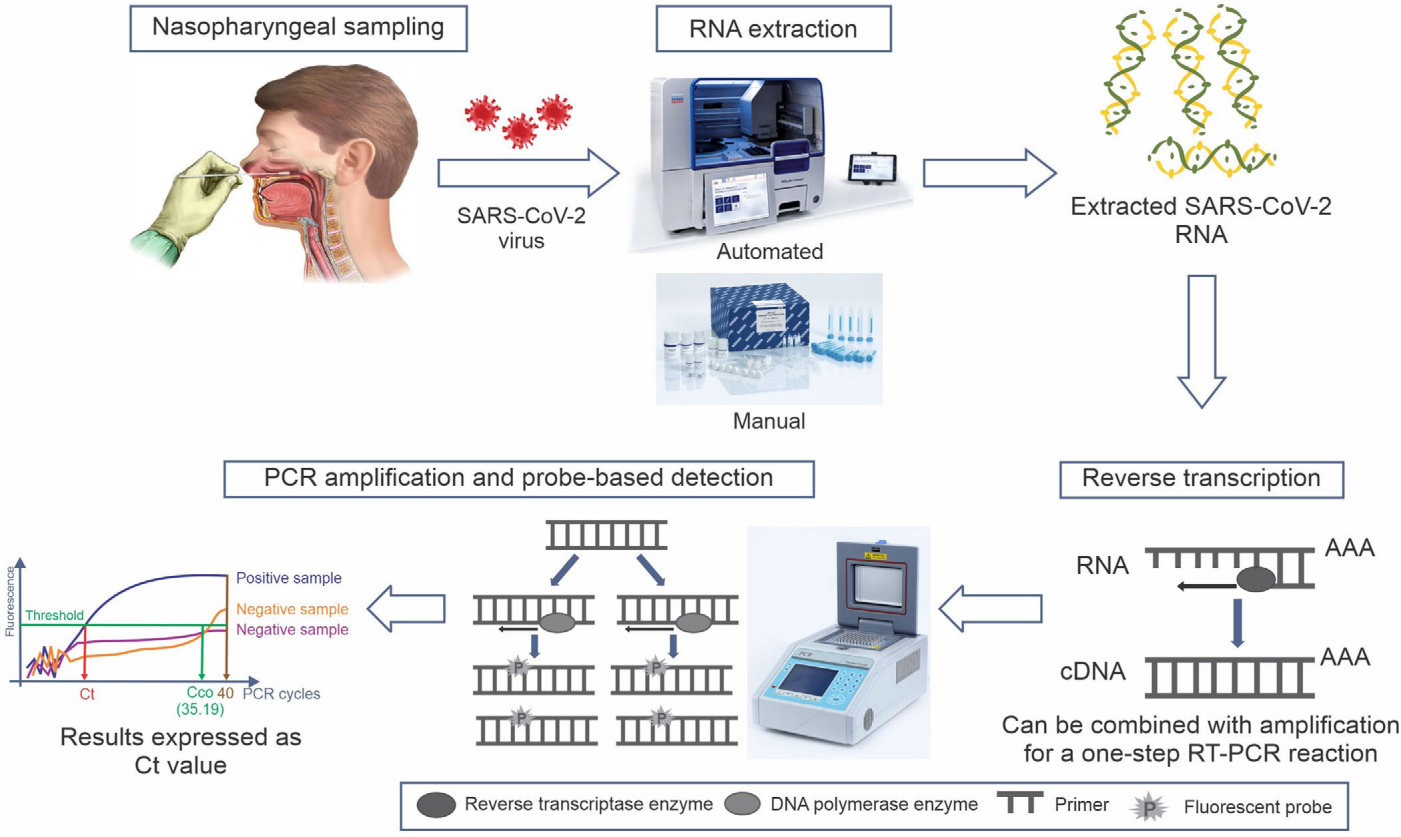
Representation of COVID‐19 FET sensor operation procedure in illustration. Redrawn with permission^70^

**FIGURE 12:**
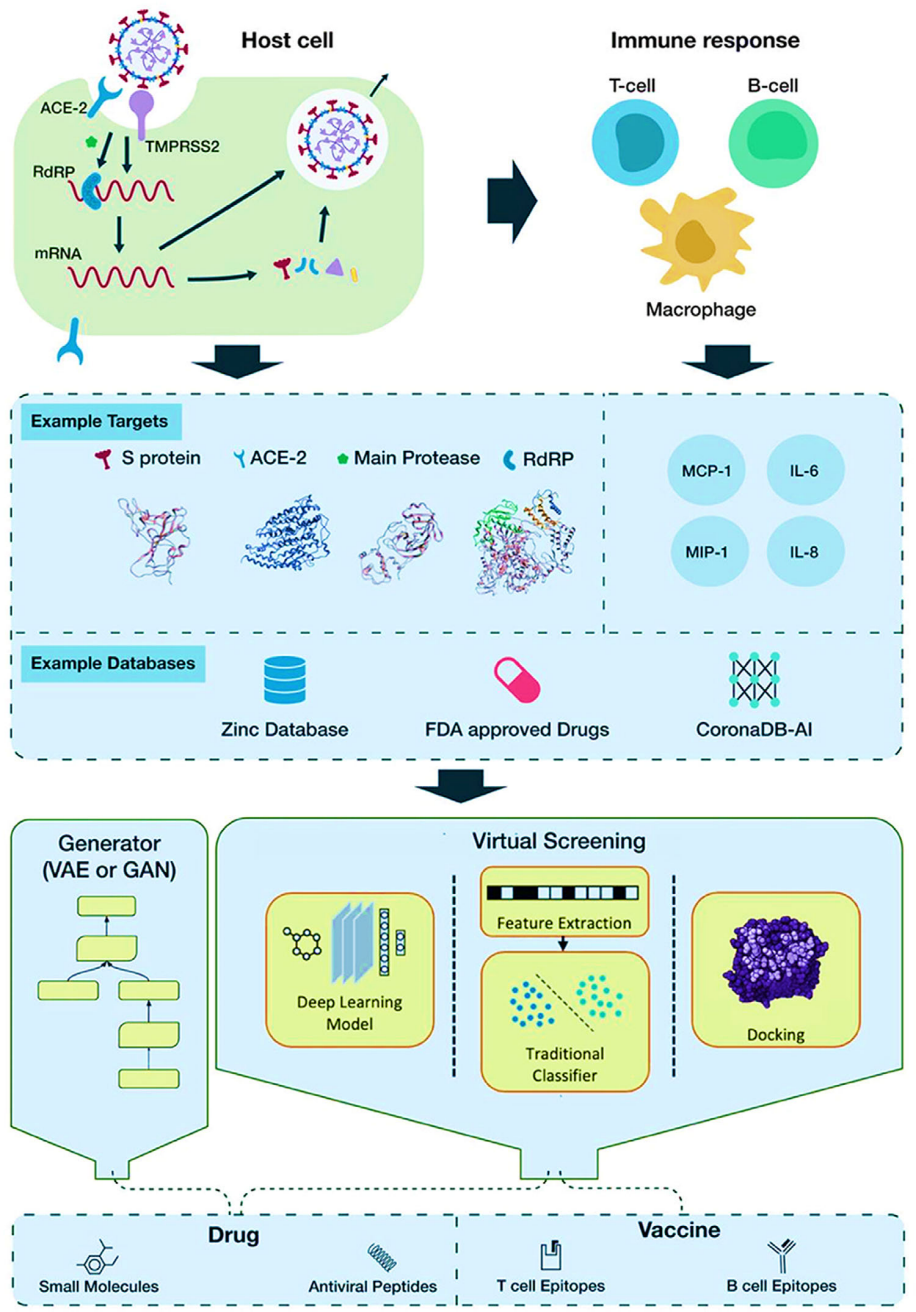
The pipeline of AI‐based drug discovery and vaccine development for COVID‐19. Reproduced with permission^87^

A field‐effect‐transistor (FET)‐based biosensor has been developed by Seo et al. for rapid detection of SARS‐CoV‐2 protein in nasopharyngeal swab (Figure 11). Here, graphene sheet was fabricated and monoclonal antibody against the spike protein is used. This sensor has shown a limit of detection of 1.6 × 101 pfu/ml using a known concentration of virus protein and 2.42 × 10^2^ copies/ml in the case of clinical samples. However, further clinical trial with justified sample size is requirement^70^. Immunosensing chip is best in production, storage, disposal, and cost effective. However, further research for nanoparticles‐based experiments required. Such sensing chip used with smartphone for easily COVID‐19 diagnostics. The Internet of things (IoT) will accomplish the bioinformatics data processing, while AI will examine the adjusted pathogenesis information. According to the patient genomic profile, all data can be analyzed together for better clinical decision (Figure 12).

### 6.3 Artificial intelligence assisted approach

Out of other technology‐based assessment, AI is one of the approaches for easily track the spread of COVID‐19 via identifying high‐risk patients with real‐time manner for the intelligent healthcare management. AI is a forthcoming tool to recognize the early infections and monitoring the real‐time condition of infected patients. This tool significantly improves the treatment regularity and resolution by developing convenient algorithm. AI has the potential to improve the treatment, planning and add impact evidence based medical‐technology.^82^, ^83^ For critically ill Covid‐19 patients, some AI‐based systems have been designed and developed for accurate prediction of COVID‐19. It is well known that COVID‐19 is associated with socio‐logical aspect disease and to overcome this issue there is a need of smart technology‐based diagnostic tools. To address these challenges, AI‐assisted machine learning, deep‐learning, and IoT approaches have vast application especially in current pandemic disease. As reported by Wang et al., Taiwan is using this type of technology as a leading disease management and to explore the big data analysis, pro‐active testing, and new technology.^84^ The results of this research and development were beneficial for appropriate diagnosis verdicts. Therefore, adaptation and implementation of technology is helpful to government public health authorities in proper action. Taiwan used both the smartphone‐based technologies and people support. This intelligent system is very important for health workers working in the root level for quick diagnostics and long‐standing follow‐up and monitoring of disease progression and health management.^85^

In current scenario, the developed COVID‐19 medicines have some additional side effects especially on heart and lungs. These recommended medicines need to modify in such a way to have a site‐specific target, which can only operative against SARS‐CoV‐2, without any side effect. To deal this issue, Zhang et al. explored the deep‐learning‐based approach COVID‐19 treatment. This approach Deeper‐Feature Convolutional Neural Network (DFCNN) deals with the RNA sequences, which are collected from the GISAID database and homology modeling has been used to explore the relevant 3D protein sequences. This approach investigated the possible ligand–protein interaction with high accuracy, without performing molecular dynamics and docking. This approach explored the ideology that peptide‐based medicine displayed negligible immune response along with desired bioavailability and stability.^86^

Figure 12 shows AI‐based drug discovery and vaccine advance models for the COVID‐19.^87^ The authors have emphasized the purpose of deep learning approach for SARS‐COV‐2 with multiple molecular targets of COVID‐19, reticence of which may improve patient endurance. Furthermore, they presented Corona DB‐AI, a dataset of epitopes, peptides, and compounds encountered either in silico or in vitro that can be hypothetically employed for exercise prototypes to obtain COVID‐19 therapy.

## 7 CONVALESCENT PLASMA THERAPY

Convalescent plasma therapy (CPT) has displayed a ray of hope, when there was no approved specific antiviral agent for COVID‐19, during this period. Some case studies have supported the hypothesis that CPT could be better treatment options.^88^, ^89^, ^90^, ^91^ CPT is a kind of passive immunity and this therapy targets at transfusing antibodies from a recovered person to an infected patient. Recently, United State of America and India have followed and suggested it. In modern‐day past, this method has effectively utilized to battle alongside the pandemic of SARS, MERS, and H1N1 influenza 2009.^92^, ^93^, ^94^ Contemporary research described the management of convalescent sera in China for the COVID‐19 affected persons.^95^

**FIGURE 13:**
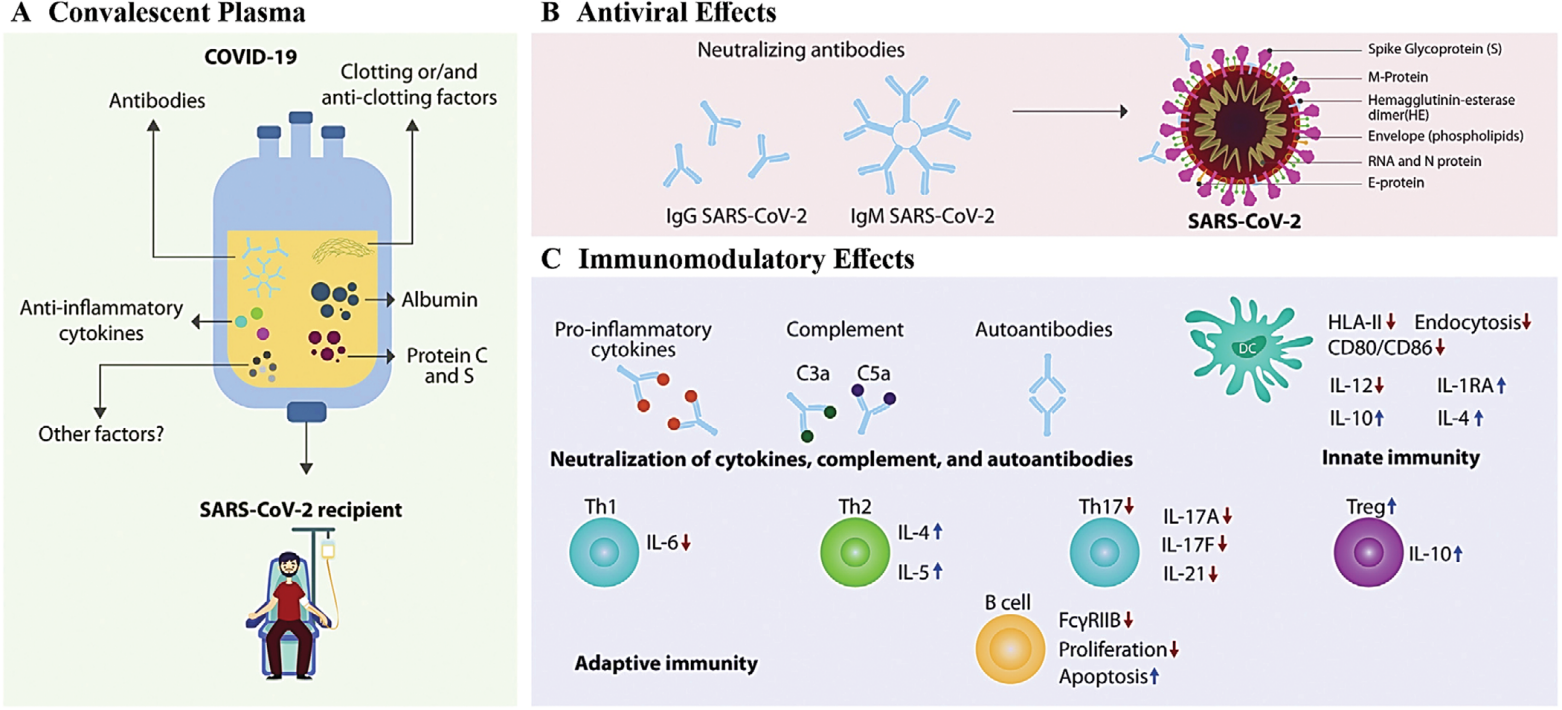
Convalescent plasma therapy. Reproduced with permission^97^

Benefactors with elevated titer quantity and suitable on additional medical factors can be acknowledged for the CP‐treatment. Cao et al. (2007)^96^ demonstrated the reducing quantities of counteracting Abs to SARS‐CoV and MERS‐CoV within a pair of months. Figure 13 characterizes the likely mechanisms of action of CP treatment and its respective consequence in COVID‐19 pathogenesis, including nullification of the virus, control on overactive immune response, and immunomodulation of hypercoagulation states. The CP treatment is expected to achieve in the hope of reducing mortality and morbidity.^97^

## 8 ROADMAP FOR EFFECTIVE TESTING AND DISEASE DIAGNOSIS

This roadmap aims to emphasize the global gap in COVID‐19 testing through the expansion of pandemic. The availability and accessibility of rapid and cost‐effective diagnostic model facilitate the better public health management.^98^, ^99^ This roadmap model addresses the need for the development of a sustainable healthcare–industry duo model for quality‐assured COVID‐19 rapid diagnostic testing.^100^ The pathway on the roadmap should integrate model referring consultative process that involved multi‐task strategies to access to COVID‐19 diagnostic testing and control. Overall, effective policy, best strategies with integration and adaptation of regional and national factors along with technology‐based monitoring and follow‐up, is the key factors addressed for diagnosis and preventive measures. Described roadmap is important for regional COVID‐19 prognosis strategies for deploying quality‐assured diagnosis services at all levels for self and community levels.^101^

On the other hand, value assessment of biomarkers, sensors technology, and advanced materials for diverse functionality and compatibility to deliver desired bioactivity is an important taskto be included in the roadmap of the public health system.^102^, ^103^, ^104^, ^105^ Potential inclusion of advanced bio‐ and nanomaterials, tissue engineering and digital technologies make substantial contribution for functional therapeutics and diagnostic devices.^98^, ^106^, ^107^, ^108^, ^109^, ^110^

Moreover, imperious to consider nanomaterials‐based biosensors research for diagnosis and therapeutics can transform the biomedical procedures for various diseases.^111^, ^112^, ^113^, ^114^ Several studies emphasize for building resilient innovative solutions to existing challenges of hospital waste management and make more better understanding for sustainable chemicals and healthcare technology for disease control. ^100^, ^101^ ^,115^.

## 9 FUTURE PROSPECTIVE

In the present COVID‐19 global health crisis, which demands the more easily accessible testing techniques and kits to overcome coronavirus diseases to save the life. There is the need of establishing wireless medicines and accurate distance screening equipment because not only those people who are encountering infectious patients but also providing safety to first line warriors, performing diagnosis and treatment to severe COVID‐19 patients. The emergence of varied coronavirus (SARS CoV‐2) pathogens will generate new challenges from time to time, even vaccine arrives. Such kind of issues could be deal with new strategies in diagnosis, drug, and vaccine research. Deep research on the continuing therapies has been effective in control. Overall, all above science and technology will control the harmful effect of COVID‐19 pandemic more effectively if we adopt the well‐defined roadmap and sustainable prognosis model. From the last decades, nanomaterial‐based biosensors encourage to generate programmable bioelectronics, while digital technologies capable of emulating the delivery of public healthcare management^100^, ^101^, ^107^, ^108^, ^109^, ^110^, ^111^, ^116^. Here, adaptation of intelligent technologies could immense boost up in the development of precision medicine to mass services.

## 10 CONCLUSIONS

During the later stage of pandemic year, tremendous progress in COVID science and technology has been made to develop for accurate and rapid diagnosis of coronavirus including the asymptomatic case. The early‐stage detection has reduced the spread of this disease in greater extent. Since December 2019, the COVID‐19 is an enduring serious global challenge, but science and technology opens a fight against COVID‐19 to deal with the human health management. While the social distancing is one of the common trends to control the spread of COVID‐19 during pandemic year, but in addition several other practices have been considered controlling the spread of this disease. Therefore, emerging technology and system to provision the operative diagnosis of COVID‐19 vital and such requirement is still welcomed by the multidisciplinary researcher to develop simple, accurate, and high‐throughput tools and techniques. However, RT‐PCR is costly, labor intensive, requires specialized laboratory equipment and time‐consuming technique but during pandemic year, this method is one of the most reliable and served as a routine in practice for the recognition of SARS‐CoV‐2.

## CONFLICT OF INTEREST

The authors declare that there is no conflict of interest.
